# Effect of Ho^3+^ Substitution on Magnetic Properties of ZnCr_2_Se_4_

**DOI:** 10.3390/ijms25147918

**Published:** 2024-07-19

**Authors:** Izabela Jendrzejewska, Tadeusz Groń, Elżbieta Tomaszewicz, Zbigniew Stokłosa, Tomasz Goryczka, Jerzy Goraus, Michał Pilch, Ewa Pietrasik, Beata Witkowska-Kita

**Affiliations:** 1 Institute of Chemistry, University of Silesia in Katowice, 40-007 Katowice, Poland; ewa.pietrasik@us.edu.pl; 2 A. Chełkowski Institute of Physics, University of Silesia in Katowice, 40-007 Katowice, Poland; tadeusz.gron@us.edu.pl (T.G.); jerzy.goraus@us.edu.pl (J.G.); michal.pilch@us.edu.pl (M.P.); 3 Faculty of Chemical Technology and Engineering, West Pomeranian University of Technology, 70-310 Szczecin, Poland; 4 Institute of Materials Science, University of Silesia in Katowice, 40-007 Katowice, Poland; zbigniew.stoklosa@us.edu.pl (Z.S.); tomasz.goryczka@us.edu.pl (T.G.); 5 Research Network Łukasiewicz—Institute of Mechanised Construction and Rock Mining, Katowice Branch, 02-673 Warszawa, Poland; b.witkowskakita@gmail.com

**Keywords:** doped spinels, holmium ions, high-temperature powder sintering, magnetic characterization, specific heat, critical fields

## Abstract

A series of ZnCr_2−*x*_Ho*_x_*Se_4_ microcrystalline spinels (where *x* = 0.05, 0.075, and 0.10) containing holmium ions in octahedral coordination were obtained by sintering of adequate reactants at high temperatures. The obtained doped materials were characterized by X-ray diffraction, Scanning Electron Microscopy, UV–Vis–NIR, molecular field approximation, and XPS spectroscopies. Their thermal properties were also investigated. The doping of the ZnCr_2_S_4_ matrix with paramagnetic Ho^3+^ ions with a content of not more than 0.1 and a screened 4*f* shell revealed a significant effect of orbital and Landau diamagnetism, a strong reduction in short-range ferromagnetic interactions, and a broadening and shift of the peak of the first critical field by simultaneous stabilization of the sharp peak in the second critical field. These results correlate well with FPLO calculations, which show that Cr sites have magnetic moments of 3.19 µ_B_ and Ho sites have significantly larger ones with a value of 3.95 µ_B_. Zn has a negligible magnetic polarization of 0.02 µ_B_, and Se induces a polarization of approximately −0.12 µ_B_.

## 1. Introduction

Magnetic resonance imaging (MRI) is a non-invasive technique with high penetration power commonly used in medical diagnostics to visualize organs and tissues. The contrast in MRI is based on the differences in the density of protons between different tissues and the differences in their longitudinal (T_1_) or transverse (T_2_) relaxation times. The sensitivity of the MRI technique is relatively low because the difference in contrast between normal and abnormal tissues is generally low. Contrast agents (CAs) are often necessary to increase such contrasts. Clinically applied MRI CAs are based on Gd^3+^ chelates or superparamagnetic nanoparticles of iron oxides. However, Gd^3+^-based nanomaterials only provide good contrasts at low magnetic fields and are less effective at high ones. MRI scanners in high magnetic fields (more than 7 T) are increasingly in demand. Therefore, new contrast agents need to be developed to improve diagnostic capabilities. Among the RE^3+^ ion family, Dy^3+^ and Ho^3+^ ions are suitable for high-field T_2_ CAs due to their significant magnetic moment without magnetization saturation at very high magnetic fields. Water-dispersed nanoparticles of dysprosium or holmium fluorides (DyF_3_ and HoF_3_), Ho^3+^-doped NaGdF_4_, or DyVO_4_ and HoVO_4_ vanadates can be used as MRI CAs in biomedicine, e.g., for brain glioma imaging [1,2].

Chromium spinels are a group of compounds that exhibit interesting properties such as colossal magnetostriction [3], the spin Jahn–Teller effect [4,5], magnetoelectric or thermoelectric effects [5,6,7,8], orbital glass [9], the coexistence of ferromagnetism and ferroelectricity [10,11], the behaviour of heavy fermions [12,13], complex spin order and spin dimerization [14,15,16], and spin-orbital fluid [17,18,19]. In these materials with a general formula of ACr_2×4_, A is a divalent nonmagnetic cation (Zn, Cd, or Hg), X is a divalent anion (O, S, or Se), and Cr^3+^ ion is in the 3*d*^3^ configuration. Chromium spinels crystallize in a normal cubic spinel, with a point group of m3m and a space group (No. 227).

The ZnCr_2_Se_4_ normal spinel is a *p*-type semiconductor with a band gap of E_g_ = 1.28 eV at room temperature [20] and is antiferromagnetic (AFM) [21,22]. Therefore, it is a desirable matrix for many spinel series [23,24,25,26,27,28]. Neutronographic studies have revealed a complex AFM order with the Néel temperature T_N_ = 20 K and dominating short-range ferromagnetic (FM) interactions, as evidenced by the large positive Curie–Weiss temperature θ = 115 K. Below the T_N_, ZnCr_2_Se_4_ has a helical spin structure with a propagation vector [001]. The spins lie in the (001) FM sheets, and the turning angle between the nearest adjacent sheets is 42° [23,29]. The magnetization measured at T = 2 K along the (001) axis shows an inflexion point in the critical field H_c1_ of about 10 kOe, characteristic of the metamagnetic transition, and a transition from a conical spin structure to the saturation state in the second critical field H_c2_ of about 65 kOe [3,30]. A spectacular transition to the AFM state is observed at temperature T_N_, accompanied by a structural transformation from cubic symmetry to an *I*4_1_*/AMD* tetragonal one with a slight contraction along the c axis of c/a = 0.9999 [31]. In 2004, in the ZnCr_2_Se_4_, the transition from positive to negative magnetoresistance was discovered [32].

The magnetic studies of the ZnCr_2_Se_4_ compound and its spinel series did not consider the contributions of magnetic susceptibility independent of temperature, the presence of which may significantly affect the correctness of the magnetic parameters. In general, the total measured magnetic susceptibility vs. temperature T is a sum of the paramagnetic Curie–Weiss susceptibility χ = C/(T − θ) (where C is the Curie constant) and the temperature-independent contributions to susceptibility (χ_0_) coming from the orbital and Landau diamagnetism, Pauli and Van Vleck paramagnetism, as well as others [19].

The main goal of this work is to investigate the effect of substituting holmium ions into the microcrystalline ZnCr_2_Se_4_ matrix on magnetic properties and critical fields, taking into account the temperature-independent contribution of magnetic susceptibility. For this purpose, specific heat measurement and local-orbital full-potential calculations were used. Our work continues previous research on RE^3+^-doped ZnCr_2_Se_4_ single crystals (RE = Nd, Gd, Dy, and Ho) [25,26,27,28].

## 2. Results and Discussion

### 2.1. Structural and Analytical Analysis

**Figure 1:**
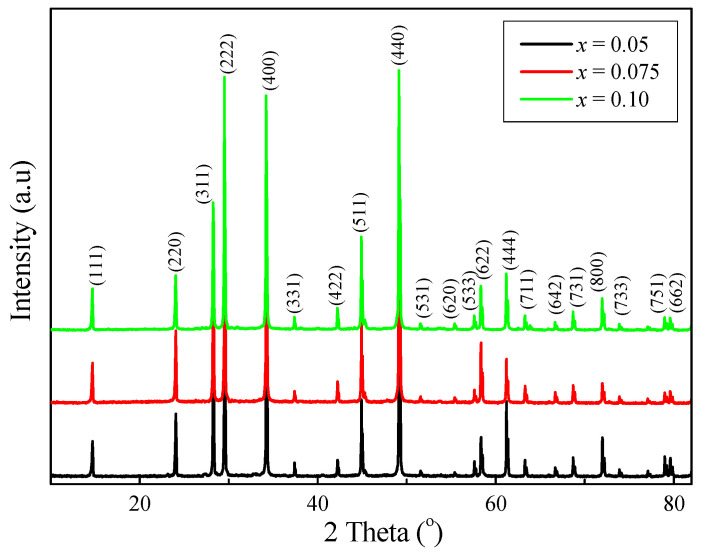
X–ray diffraction patterns of ZnCr_2−*x*_Ho*_x_*Se_4_ samples (*x* = 0.05, 0.075, and 0.10).

**Figure 2:**
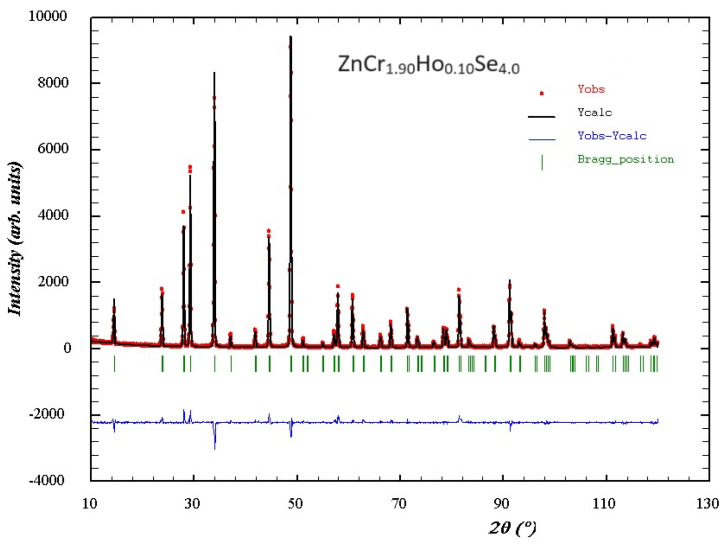
The relation between observed and calculated X–ray diffraction patterns and their difference for the ZnCr_2−*x*_Ho*_x_*Se_4_ sample when *x* = 0.10.

Powder XRD patterns of ZnCr_2−*x*_Ho*_x_*Se_4_ samples with different Ho^3+^ ion contents, i.e., when *x* = 0.05, 0.075, and 0.10, are shown in Figure 1. The XRD studies revealed that these patterns consisted of diffraction peaks that can be attributed to the spinel framework only. No other phases and initial reactants, i.e., ZnSe and Cr_2_Se_3_, were observed in the ZnCr_2−*x*_Ho*_x_*Se_4_ solid solution. The observed diffraction lines attributed to a spinel-type structure shifted towards the lower 2Theta angle with increasing Ho^3+^ concentration. This is typical behaviour when smaller Cr^3+^ ions in the ZnCr_2_Se_4_ matrix are substituted by much larger Ho^3+^ ones (Table 1). Rietveld analysis was applied to the doped samples using the FullProf programme, v.3.40 for Windows [33,34]. The obtained results are presented in Tables S1–S3 and Figure 2 for the ZnCr_1.90_Ho_0.10_Se_4_ sample.

The results of Rietveld refinement confirmed that the obtained samples crystallized in a cubic, spinel-type structure with space group *Fd3m* (ZnCr_2_Se_4_, ICDD PDF2, No. 03-065-0689).

**Figure 3:**
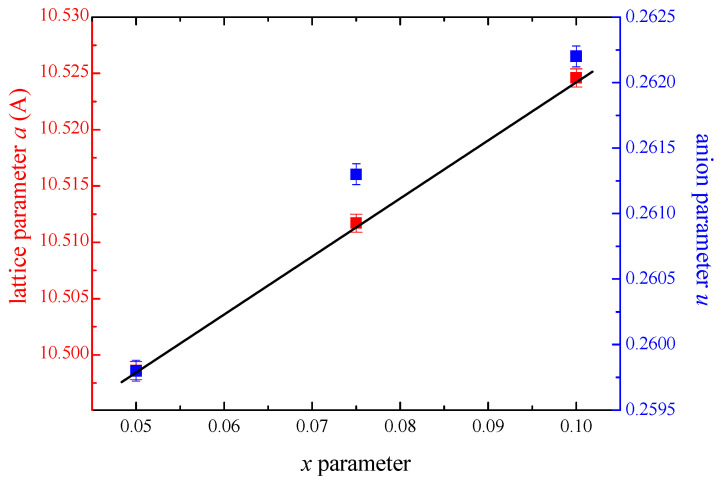
The dependence of the lattice parameter (a) and the anion parameter (u) of ZnCr_2−x_Ho_x_Se_4_ on the concentration of holmium ions.

The unit cell parameter (*a*) of ZnCr_2−*x*_Ho*_x_*Se_4_ samples increases almost linearly as the concentration of Ho^3+^ ions increases, thus fulfilling Vegard’s law (Figure 3). The anion parameter (*u*) is also changed similarly (Figure 3). The unit cell parameters, refinement parameters, atomic coordinates, and occupancy factors (*g*), as well as bond lengths, are given in Tables S1–S3, respectively. The relation between observed and calculated X-ray diffraction patterns and their difference for the ZnCr_2−*x*_Ho*_x_*Se_4_ sample when *x* = 0.05 and 0.075 are presented in Figures S1 and S2. The occupancy factors for chromium ions decrease as the content of Ho^3+^ ions increases. The change in lattice parameters and occupancy factors confirms that Ho^3+^ ions partially substituted Cr^3+^ ones in the ZnCr_2_Se_4_ matrix.

The SEM-EDX technique was used to determine the chemical composition of the obtained samples. Each microcrystalline sample was measured at 20 different points. These results, presented in Table 2, confirmed the assumed formulas. The EDX spectra for all samples are presented in Figures S3–S5.

The intensities of diffraction lines depend directly on the ion location in the spinel elementary cell. The distribution of cations for the ZnCr_2−*x*_Ho*_x_*Se_4_ solid solution was obtained by the Bertaut method [38]. This method compares the ratio of calculated diffraction line intensities with the values observed in the experimental diffraction pattern. Several pairs of reflexes were matched according to Equation (1):

(1) $$\frac{I_{hkl}^{obs}}{I_{h^{\prime }k^{\prime }l^{\prime }}^{obs}}=\frac{I_{hkl}^{cal}}{I_{h^{\prime }k^{\prime }l^{\prime }}^{cal}}$$

where the observed and calculated intensities for the *hkl* diffraction line are given, respectively. The best values were obtained when comparing the ratios of the experimental and calculated values of reflexes, for which the intensities (1) are independent of the anionic parameter, (2) change together with the distribution of cations, and (3) do not differ significantly. Lines with Miller indices *220*, *400*, *440*, *422*, and *444* were used to calculate the intensity ratio, as they are most sensitive to the distribution of cations [39]. If the coefficient of convergence R, expressed by Formula (2):

(2) $$R=\left|\left(\frac{I_{hkl}^{obs}}{I_{h^{\prime }k^{\prime }l^{\prime }}^{obs}}\right)-\left(\frac{I_{hkl}^{cal}}{I_{h^{\prime }k^{\prime }l^{\prime }}^{cal}}\right)\right|,$$

reaches a minimum value, the best fit between the theoretical and real structure is obtained [38]. The observed intensities originating from planes *220*, *440*, and *311* were used to determine the degree of orientation. The degrees of orientation *hk0* (O_hk0_) and 220 (O_220_) were determined from the formulas I_220_ + I_440_/I_311_ and I_220_/I_311_, respectively [39]. The results of the calculations of intensity ratios of the corresponding diffraction lines and the degree of orientation are gathered in Table S4. A perfect ratio conformity between the observed and the calculated intensities suggests that the actual distribution of cations in the sublattices is in good accordance with the assumed model—ZnCr_2−*x*_Ho*_x_*Se_4_. Higher values of O_hk0_ and O_220_ allow for assuming that most crystallites are oriented in directions [hk0] and [220], respectively. The values of O_hk0_ and O_220_ indicate a lack of preferred orientation. Crystallites are oriented in a statistically random way. Table S4 shows consistent cation distributions obtained from the experiment and the computational method.

The increase in Ho^3+^ ion concentration causes an increase in the average cation ionic radius in the tetrahedral position (*<*R_A_*>*) and in the octahedral position (*<*R_B_*>*) for ZnCr_2−*x*_Ho*_x_*Se_4_. Such a change in the average ionic radius causes an increase in the deformation of the anion octahedron and in the A–X distances. This tendency leads to the minor stabilization of the ionic bond of Cr^3+^ ions and an increase in the ionic bond contribution in the tetrahedral sublattice [40]. The ion packing coefficients P_t_ and P_o_ have been calculated based on the equations presented in Ref. [41]. The small values of P_t_ and P_o_ (<1.0) corroborate the smaller ion distances and the more extensive overlap of the cation and anion orbitals. They also corroborate the likelihood that the tetrahedral and octahedral positions will be empty, indicating the presence of ion vacancies [40,41]. In the ZnCr_2−x_Ho_x_Se_4_ solid solution, as the holmium ions content increases, the P_t_ coefficient values increase, the P_o_ coefficient values decrease, and the calculated parameter increases compared to pure ZnCr_2_Se_4_ matrix (Table S4).

**Figure 4:**
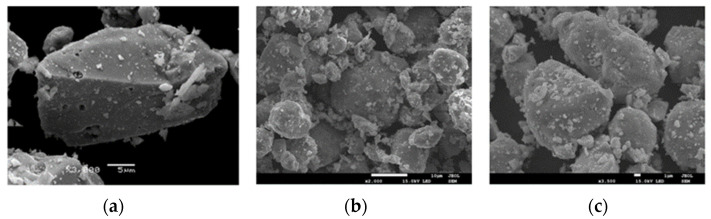
A fracture surface by SEI for ZnCr_2−x_Ho_x_Se_4_ samples, scale bar = 1 μm. (**a**) ZnCr_1.95_Ho_0.05_Se_4.0_, magnification 3000×, (**b**) ZnCr_1.925_Ho_0.075_Se_4.0_, magnification 2000×, and (**c**) ZnCr_1.90_Ho_0.10_Se_4.0,_ magnification 3500×.

**Figure 5:**
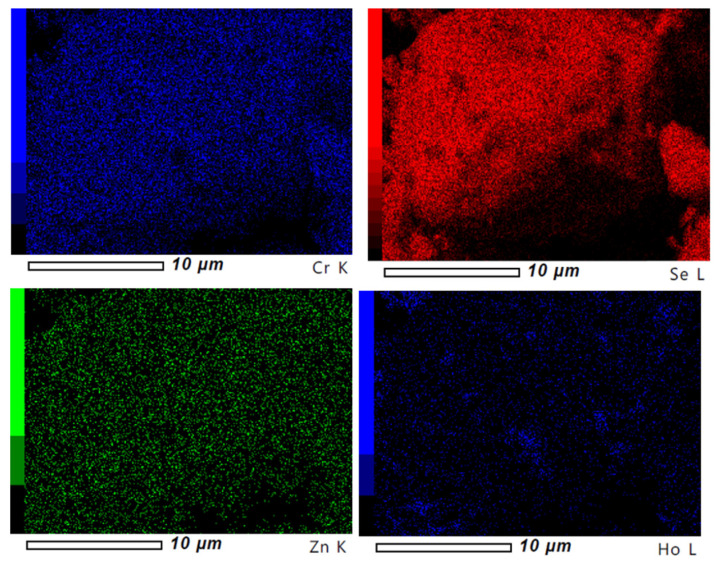
Map of element distribution measured for ZnCr_1.90_Ho_0.10_Se_4.0_ sample.

Scattered electron images (SEIs) were collected at room temperature. High-magnification SEM microphotographs revealed the grainy structure of obtained samples with visible, oval-shaped single microcrystals (Figure 4). Scanning microstructure analysis indicates a regular distribution of all elements throughout the sample volume (Figure 5).

### 2.2. Thermal Stability

**Figure 6:**
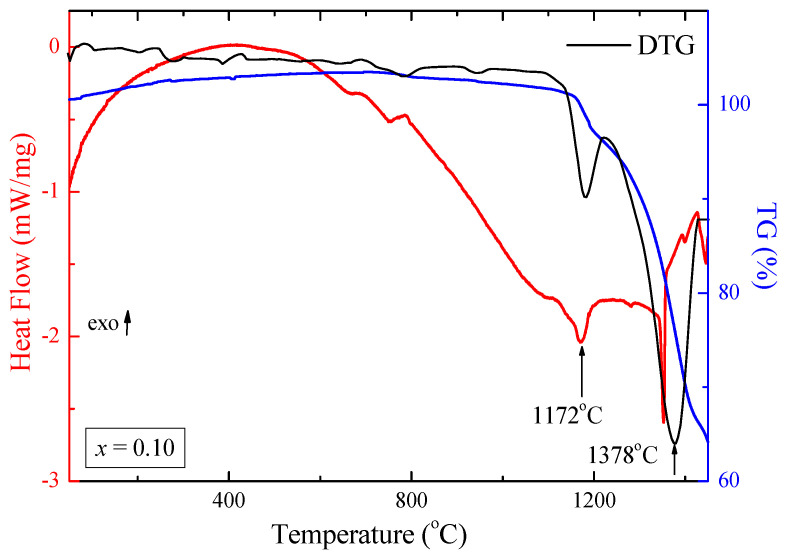
DSC–TG–DTG curves recorded during controlled heating of the ZnCr_1.90_Ho_0.10_Se_4.0_ sample.

The results of DSC-TG measurements are presented in Table 3 and Figure 6 (for the sample with *x* = 0.10). The mass loss is much more significant than pure ZnCr_2_Se_4_ and equals about 36 mass% for ZnCr_1.90_Ho_0.10_Se_4.0_ (Table 3).

For the samples doped with holmium ions, the melting process is observed at higher temperatures, i.e., at ~1150 °C, in comparison to pure ZnCr_2_Se_4_ (at ~725 °C) (Table 3). The small concentration of Ho^3+^ ions in the crystal lattice of ZnCr_2_Se_4_ causes an increase in the share of ionic binding and a higher melting temperature because the Ho^3+^ ion has a larger ionic radius than Zn^2+^ and Cr^3+^ ones; its electronegativity is also much lower than other metallic elements in ZnCr_2−*x*_Ho*_x_*Se_4_ (Table 1). A similar phenomenon was observed in Zn*_x_*Mn*_y_*Cr*_z_*Se_4_ [24].

Meanwhile, for the sample with a higher amount of holmium ions (*x* = 0.1), the melting of samples starts at a lower temperature, and the process of degradation occurs in several stages. For the ZnCr_2−*x*_Ho*_x_*Se_4_ samples, three peaks are observed in the DTG curve at 553 °C, 832 °C, and 1162 °C. The melting process of this sample occurs in three steps. This phenomenon indicates that the more significant amount of Ho^3+^ ions causes a decrease in the share of ionic bonds and an increase in the share of covalent bonds due to the weakening of the crystal lattice.

As shown in Table 4, the thermal parameters confirmed that holmium affects doped compounds’ thermal stability and resistance compared with the pure ZnCr_2_Se_4_.

### 2.3. FPLO Calculation

**Figure 7:**
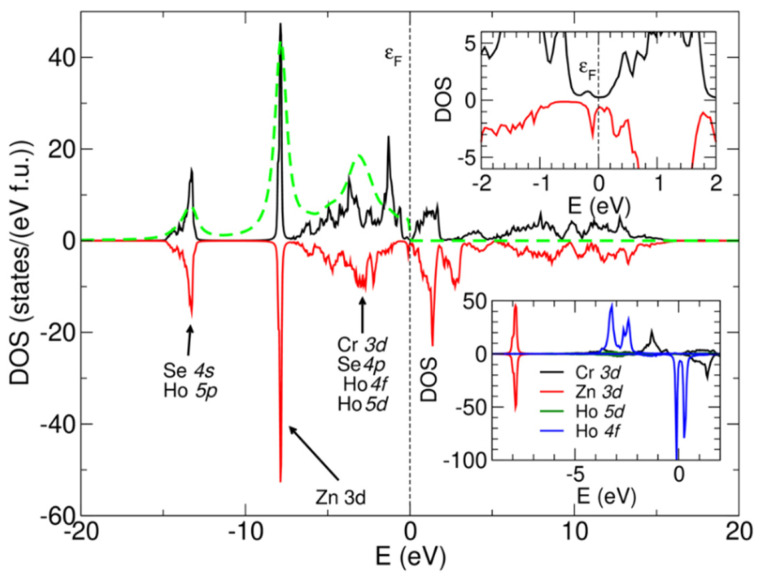
The total density of states calculated for the ZnCr_1.95_Ho_0.05_Se_4.0_ sample.

Figure 7 shows the calculated densities of states for the ZnCr_1.95_Ho_0.05_Se_4.0_ sample (main figure; two spin channels). The dashed line shows the simulated X-ray Photoemission Spectroscopy (XPS) spectrum for the photon energy 1486.3 eV (Al K alpha line), which was obtained by multiplication of the corresponding partial densities of states by the photoionization cross-sections taken from [42] and convoluted with Lorentz (FWHM = 0.4 eV) and Fermi–Dirac functions (room temperature was assumed). The highest contribution to the simulated XPS spectrum was observed for Se 4*s* and Ho 5*p* states (13 eV region); Zn 3*d* states (8 eV); and Cr 3*d*, Se 4*p*, Ho 5*d,* and Ho 4*f* states (2–6 eV below the Fermi level ε_F_).

The unsubstituted ZnCr_2_Se_4_ spinel exhibits a half-metallic electronic structure with a band gap in one spin channel and metallic density of states in the other channel. In the substituted compound, additional states emerge in the gap, visible in the upper inset of Figure 7. The lower inset of Figure 7 shows the contribution of *d* states of transition metals and 4*f* and 5*d* states of Ho to the total density of states. It is visible that the Zn *d* states do not exhibit magnetic order, whereas the Cr and Ho states do. The minimum in the density of states is 0.2–0.3 eV width at the Fermi level—this is to be expected for two reasons: (i) Substitution, apart from the modifications and shifts in band structure, usually creates disorder and additional states in the bandgap. (ii) The LSDA potential [43,44] is well known to underestimate the bandgaps. Therefore, we fully expected the experimentally determined bandgap to be larger.

Calculations show that Cr sites exhibit magnetic moments of 3.19 µ_B_, whereas Ho sites exhibit 3.95 µ_B_. Zn has negligible magnetic polarization of 0.02 µ_B_, whereas Se has induced polarization of about −0.12 µ_B_. In addition, Mulliken charge analysis [45] shows that Cr and Ho have similar charges (+0.8e and 0.89e, respectively) within the Cr site, which is being substituted. Zn has a lower charge (+0.519e) than Cr, and Se has −0.529e. The magnetic moment and charge on Cr sites are similar to other substituted ZnCr_2_Se_4_ solid solutions (see our earlier works).

### 2.4. UV–vis–NIR Studies

Diffuse Reflectance Spectroscopy is the standard method used in the determination of absorption properties of solid materials. Optical parameters such as the absorption coefficient, band gap energy, and refractive index are important parameters that characterize materials for their various industrial applications. Band gap energy is also the primary factor determining the electrical conductivity of solids.

When a semiconductor absorbs photons of energy larger than its gap, an electron is transferred from the valence band to the conduction band, and a sharp increase in the absorption of the material to the wavelength corresponding to the band gap energy occurs. The relation of the absorption coefficient (α) to the incidental photon energy (hν) depends on the type of electronic transition. Two types of optical transitions can occur at the fundamental edge of crystalline materials: direct and indirect transitions [46,47,48]. For materials with a direct band gap, valence electrons can be directly excited into the conduction band by a photon whose energy is larger than a band gap. Indirect transition also involves simultaneous interaction with lattice vibration.

**Figure 8:**
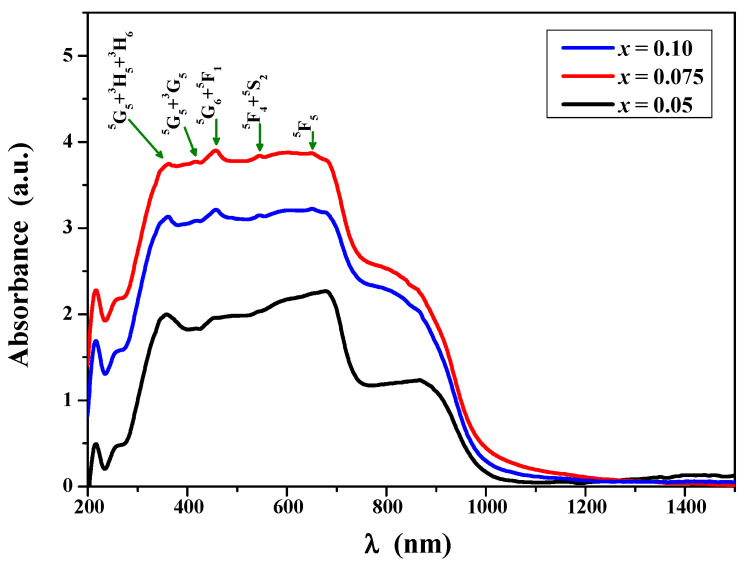
UV–vis–NIR absorption spectra of ZnCr_2−*x*_Ho*_x_*Se_4_ when *x* = 0.05; 0.075; and 0.10.

Figure 8 shows UV–vis–NIR absorption spectra recorded under ambient conditions for ZnCr_2−*x*_Ho*_x_*Se_4_ (x = 0.05; 0.075 and 0.10) solid solution. All samples show energy absorption ability in the ultraviolet, visible, and near-infrared ranges. This is confirmed by the wide absorption bands recorded in these wavelength ranges. On the other hand, the low-intensity bands in the visible region observed at around 360, 420, 455, 545, and 650 nm can be attributed to the transitions from the ground state ^5^I_8_ of Ho^3+^ ion to the excited states: ^5^G_5_ + ^3^H_5_ + ^3^H_6_, ^5^G_5_ + ^3^G_5_, ^5^G_6_ + ^5^F_1_, ^5^F_4_ + ^5^S_2_, and ^5^F_5_ [49,50]. In this work, we focused only on absorption spectra analysis to determine the energy gap and the type of electronic transition in the samples under study.

**Figure 9:**
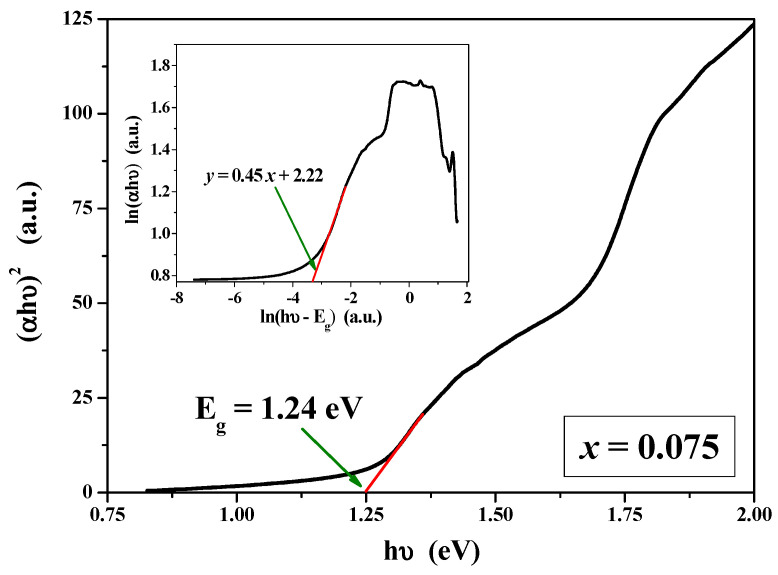
The plot of (αhν)^2^ vs. hν of ZnCr_2−*x*_Ho*_x_*Se_4_ when *x* = 0.075 with determined direct optical band gap (E_g_). Insert: plot of ln(αhν) vs. ln(hν − E_g_) of ZnCr_2−*x*_Ho*_x_*Se_4_ when *x* = 0.075.

The optical band gap (E_g_) can be determined using the Tauc and Wood relation [46,47,48]:

αhν = A(hν − E_g_)^n^ (3)

where *A* is an energy-independent constant characteristic of a material and *n* is a constant that can take different values depending on the nature of the electronic transition. The permitted direct and indirect transitions occur when *n* = 1/2 and 2, respectively [47,48,49]. The values of E_g_ were determined considering the direct transition from the plot of (αhν)^2^ as a function of hν by extrapolating the linear portion of the curve to the photon energy axis at zero absorption (Figure 9) [51,52,53].

The band gaps of ZnCr_2−*x*_Ho*_x_*Se_4_ samples were 1.22 eV when *x* = 0.05 and 1.24 eV when *x* = 0.075 and 0.10. The observed values of E_g_ are slightly lower than the optical band gap (1.28 eV) determined for a single crystal of pure matrix, i.e., ZnCr_2_Se_4_ [21]. To further confirm that obtained ZnCr_2−*x*_Ho*_x_*Se_4_ materials are direct band gap semiconductors, the Tauc and Wood equation was transformed into the following form [53]:

ln(αhν) = lnA + n ln(hν − E_g_)^n^ (4)

where *n* is the slope of the ln(αhν) vs. ln(hν − E_g_) relation. The insert in Figure 9 shows a plot of ln(αhν) vs. ln(hν − E_g_) for a sample of solid solution when *x* = 0.075. The linear fit of this curve gives a slope value of 0.45, which is nearly one-half. It means that the obtained Ho^3+^-doped materials show a direct allowed band gap.

### 2.5. Specific Heat Capacity

**Figure 10:**
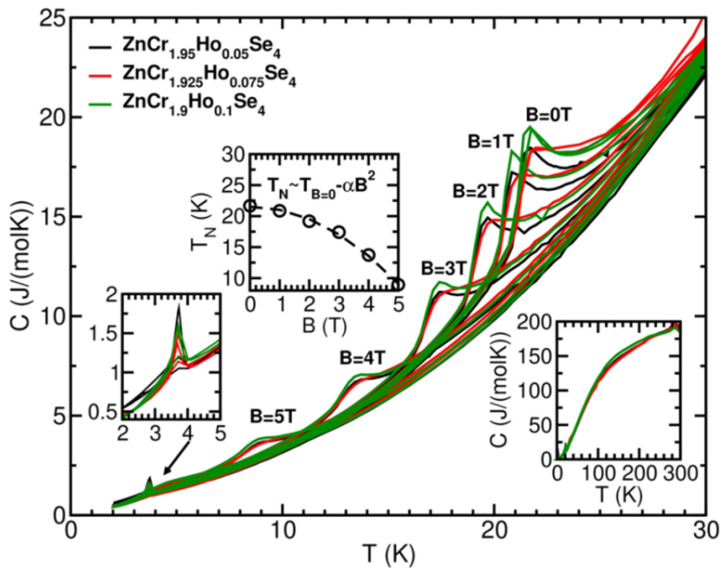
Heat capacity measured in the narrower temperature range around the magnetic transition (main figure) and in the broad temperature range (right bottom inset). The left inset shows a low-temperature peak at 3.8 K, observed for all samples. The middle inset shows the change in the magnetic peak position concerning the applied magnetic field. A quadratic dependence is noticeable here (usually for simple magnets, which the mean-field model can explain).

Three samples, ZnCr_1.95_Ho_0.05_Se_4.0_, ZnCr_1.925_Ho_0.075_Se_4.0_, and ZnCr_1.9_Ho_0.1_Se_4.0_, were measured within 2–300 K temperature and at magnetic fields 0, 1, 2, 3, 4, 5, and 6 T below 30 K. In the bottom-right inset of Figure 10, we show the results of the measurements in the wide temperature range. Due to only minute stoichiometry changes, the results of the samples under study are almost the same. At room temperature, the specific heat of the samples reaches the expected Dulong–Petit law value: 7 × 3R = 168 J/(mol K) (number of atoms times three gas constants). This shows that the sample composition was correct.

The main figure (Figure 10) shows the change in the position of the magnetic peak with magnetic field and Ho^3+^ concentration. With increasing field, the peaks are located at lower and lower temperatures, characteristic of antiferromagnetic ordering. The differences in the ordering temperatures between the samples are minute. However, the ZnCr_1.925_Ho_0.075_Se_4_ sample has a slightly different shape of the magnetic peaks than the remaining two samples. It shows that the magnetic behaviour of the samples is not monotonic with increasing Ho^3+^ concentration. The magnetic peak at 6 T is generally not visible for all samples. The peak position change concerning the magnetic field is shown in the middle inset. The change in ordering temperature with the magnetic field follows quadratic dependence, T_N_ = T(B = 0 T) − α × B^2^ (α = 0.49 K/T^2^ and T(B = 0 T) = 21.51 K), which is to be expected for a simple ferro- or antiferromagnet in a mean-field model (the differences between peak positions for the different samples are negligible, here). At about 3.7–3.8 K, a small peak is observed for all three samples, which does not shift significantly with the magnetic field and is almost quenched by a field of 1 T. It can occur in some superconducting or weak magnetic phases. Stoichiometric Ho_2_Se_3_ has an ordering temperature of about 6 K [54].

### 2.6. Magnetic Properties

**Figure 11:**
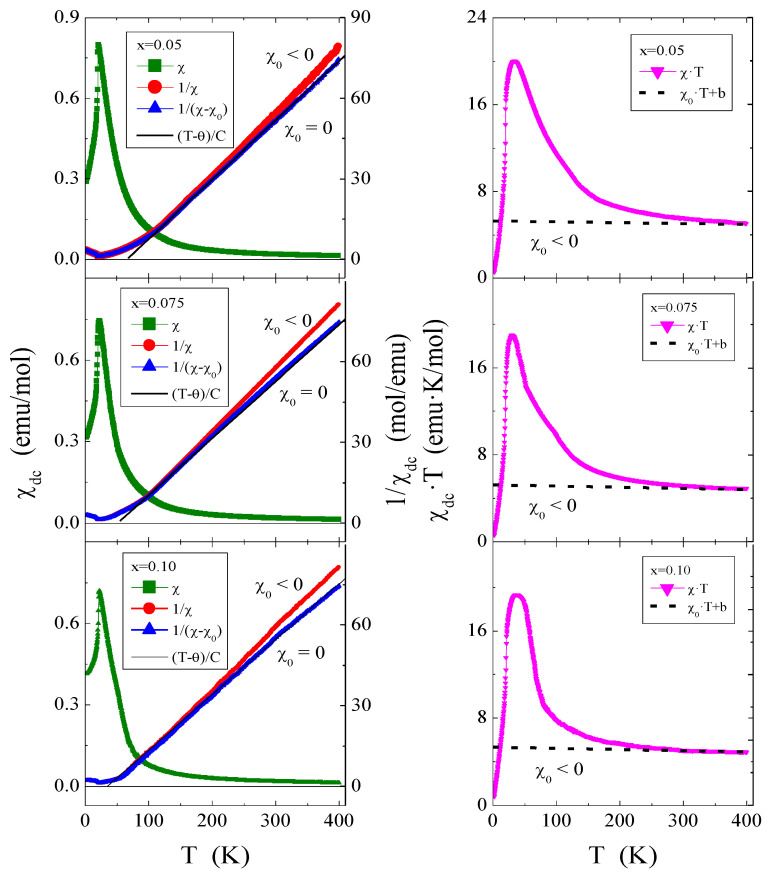
DC magnetic susceptibility χ_dc_, 1/χ_dc,_ and 1/(χ_dc_ – χ_0_) and product χ_dc_·T as a function of temperature T of a series of microcrystalline ZnCr_2−*x*_Ho*_x_*Se_4_ spinels for *x* = 0.05, 0.075, and 0.10, recorded at H_dc_ = 1 kOe. The solid, (T − θ)/C, and dashed, χ_0_·T + b, lines indicate Curie–Weiss behaviour. χ_0_ is the slope and b is the intercept.

**Figure 12:**
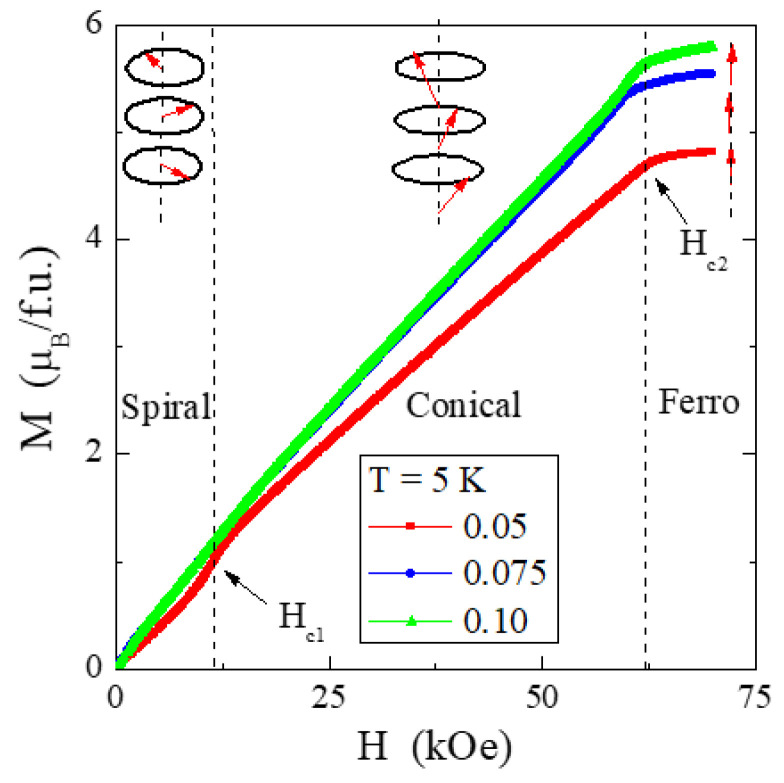
Magnetization M vs. magnetic field H recorded at 5 K of a series of polycrystalline ZnCr_2−*x*_Ho*_x_*Se_4_ spinels for *x* = 0.05, 0.075, and 0.10. The upper pictures show the evolution of magnetic structure in an external magnetic field from the spiral via conical to the FM order.

**Figure 13:**
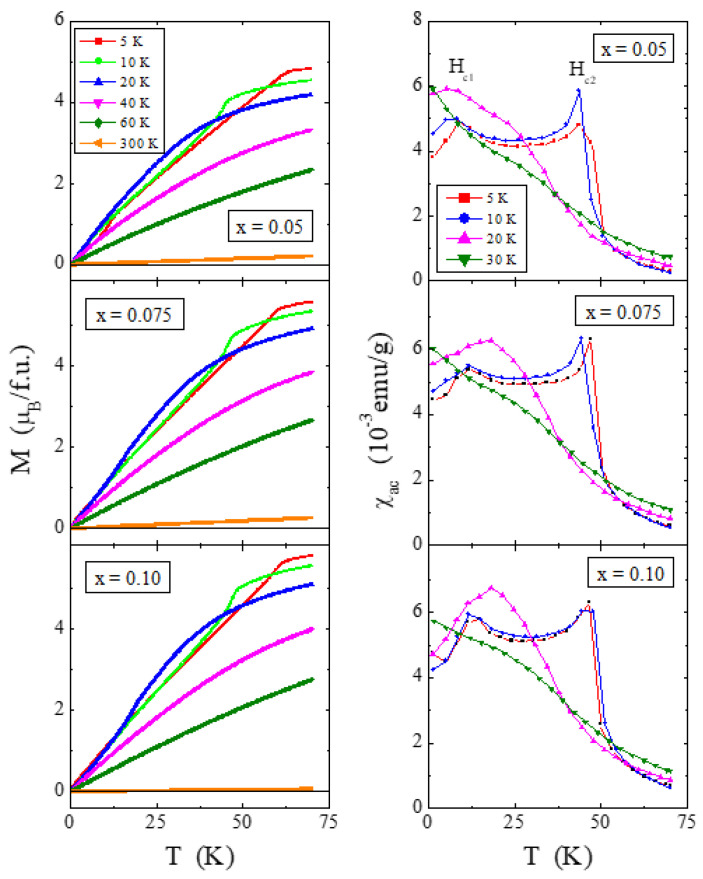
Magnetization M and ac magnetic susceptibility χ_ac_ as a function of dc magnetic field H recorded at 5, 10, 20, 40, 60, and 300 K and 5, 10, 20, and 30 K (in an internal oscillating magnetic field H_ac_ = 3.9 Oe and internal frequency f = 1 kHz), respectively, of a series of polycrystalline ZnCr_2−x_Ho_x_Se_4_ spinels for *x* = 0.05, 0.075, and 0.10.

The results of magnetic measurements of ZnCr_2−*x*_Ho*_x_*Se_4_ powder spinels are shown in Figure 11, Figure 12 and Figure 13 and in Table 4. Due to the low content of magnetic holmium ions, not exceeding *x* = 0.1, and a significant decrease in the magnetic moment for single crystals [26], in the case of the studied microcrystalline samples, it was necessary to estimate the temperature-independent magnetic contributions. In general, these contributions significantly change the value of the magnetic susceptibility in the Curie–Weiss region by the factor χ_0_, which represents all temperature-independent susceptibilities, i.e., the orbital and Landau diamagnetism, Pauli and Van Vleck paramagnetism, and others. The χ_0_ factor was determined from the linear function χT(T) [51,55,56] in the Curie–Weiss region:

(5) $$\chi T=b+\chi_{0}T$$

where b is the intercept and χ_0_ is the slope. The dependencies of χT(T) are shown in Figure 11, and the parameters b and χ_0_ are collected in Table 4. The right side of Figure 11 shows that the linear approximation of the Curie–Weiss region (dashed line) gives a negative contribution (χ_0_ < 0) and, therefore, orbital and Landau diamagnetism for all polycrystalline samples. The χ_0_ is slightly smaller than for the matrix (Table 4). The product of χT versus T in Figure 11 achieves zero, decreasing continuously upon cooling, confirming fully compensated AFM interactions between the metal centre below T_N_.

The temperature dependencies of magnetic susceptibility, χ(T), and its inverse, 1/χ(T), after taking into account the χ_0_ factors, are shown on the left side of Figure 11, and the magnetic parameters calculated in this way are summarized in Table 4. The χ_0_ factor does not significantly impact the long-range magnetic interactions visible in the stable values of the Néel temperature T_N_ and the superexchange integral J_1_ for the first coordination sphere. However, χ_0_ has a significant influence on the slope of the lines (T − θ)/C (solid line), the effective magnetic moment µ_eff,_ and the superexchange integral J_2_ for the second coordination sphere. As the content of screened paramagnetic holmium 4*f* ions increases, we observe a strong decrease in the positive paramagnetic Curie–Weiss temperature, which measures the strength of short-range FM interactions. The weakening of these interactions is also visible in the values of the superexchange integral J_2_. Replacing the Cr^3+^ ion with a smaller radius with the Ho^3+^ one with a larger radius [36] (Table 1) increases both the size of the unit cell (Table S1) and the length of the B-B bonds (Table S3), which consequently leads to reduced short-range FM interactions (Table 4) above T_N_. The experimental effective magnetic moment µ_eff_ of these spinels (see Table 4) is slightly lower than the effective Bohr magneton number p_eff_ for high-spin Cr^3+^ (S = 3/2, g = 2) and Ho^3+^ (J = 8, *g* = 5/4). This slight deviation from the ideal value can be attributed to non-stoichiometry and spin defects.

The magnetic parameters of the ZnCr_2_Se_4_:Ho single crystals of similar composition [14], in which the χ_0_ factors were not considered, differ significantly from the magnetic parameters of the microcrystalline samples under study.

Analysis of the product of χT as a function of T (Figure 11) showed that the AFM interactions are fully compensated, but the magnetic structure below T_N_ is very complex. This structure is schematically illustrated in Figure 12 for the three studied samples. It consists of a straight spiral at T = 5 K, which in the critical field H_c1_ of about 9.7 kOe transforms into a conical spiral to reach the state of FM saturation in the critical field H_c2_ of about 59.5 kOe. The presence of paramagnetic holmium ions causes a slight weakening of H_c1_ and a stronger H_c2_. The magnetic isotherms presented in Figure 13 (M vs. H) show that with an increase in the content of holmium ions, the magnetization increases despite a decrease in the content of chromium ones. It is because, according to FPLO calculations, holmium ions have a much larger magnetic moment than chromium ones. The magnetization isotherms, M(H), above T_N_ in Figure 13 are characteristic of a Brillouin curve, showing a paramagnetic response. The linear dependence of M(H) at 300 K is typical for paramagnets.

The right side of Figure 13 shows, in the H_ac_(H) curves, the evolution of the critical fields H_c1_ and H_c2_ with increasing temperature. A broadening and shift of the H_c1_ peak towards higher magnetic fields and stabilization of the sharp H_c2_ peak are observed. Compared to the ZnCr_2_Se_4_ matrix [24,25], the evolution of H_c1_ and H_c2_ is entirely different, because with increasing temperature, the intensity of the broad peak in the critical field H_c2_ drops rapidly and shifts towards lower magnetic field values. In contrast, the sharp peak at H_c1_ only slightly shifts backward. In both cases, above the ordering temperature, both critical fields disappear. In the case of the studied spinels, the influence of holmium ions on the critical fields is significant.

### 2.7. Molecular Field Approximation

**Figure 14:**
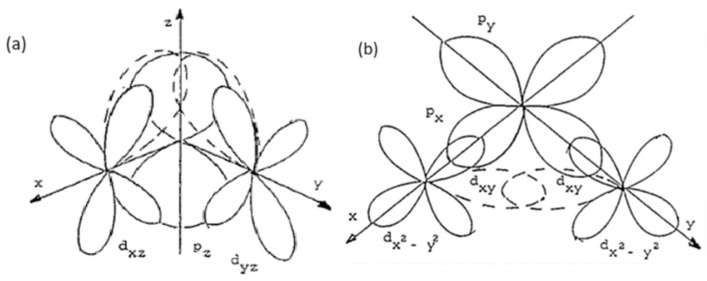
Ninety-degree exchange interaction: (**a**) with one p orbital: $d_{xz}\underline{\pi }p_{z}\underline{\pi }d_{yz}$ and (**b**) with two p orbitals: $d_{x^{2}-y^{2}}\underline{\pi }p_{x}$ and $p_{y}\underline{\sigma }d_{x^{2}-y^{2}}$. Dashed lines indicate the overlapping orbitals.

In the ZnCr_2_Se_4_ spinel below the Néel temperature T_N_ = 21.9 K (Table 4), the high and positive value of the Curie–Weiss temperature θ = 55 K results from the ferromagnetic short-range interactions of the magnetic moments of Cr^3+^ ions in the (001) planes. Each of these planes is treated as a separate subarray in the molecular field approximation with spontaneous magnetization induced by the effective molecular field representing the exchange interactions [57]. Comparable values of the effective moment and effective Bohr magneton number suggest that only the chromium ions in the 3*d*^3^ electron configuration couple ferromagnetically in the (001) planes, antiferromagnetically between the nearest planes (J_1_ < 0, Table 4), and ferromagnetically between the next nearest ones (J_2_ > 0, Table 4). The holmium ions, Ho^3+^, in the 4*f*^11^6s^2^ electron configuration, are paramagnetic; the electrons in the 4*f* orbital are strongly screened and do not participate in magnetic interactions. Magnetically interacting electrons occupy orbitals *d*_xy_, *d*_xz_, and *d*_yz_, while $d_{x^{2}}$ and $d_{x^{2}-y^{2}}$ remain empty (Figure 14). If one assumes that ligand atoms are arranged along the *z*-axis, then *d*_yz_ and *d*_xz_ orbitals overlap the p_x_ and p_y_ orbitals of the ligands. Two types of coupling between the t_2g_ states of two chromium ions through a ligand atom can appear and are called π and σ. Overlapping orbitals in the studied spinel series reduce the energy gap and significantly influence electronic transport.

### 2.8. XPS Studies

**Figure 15:**
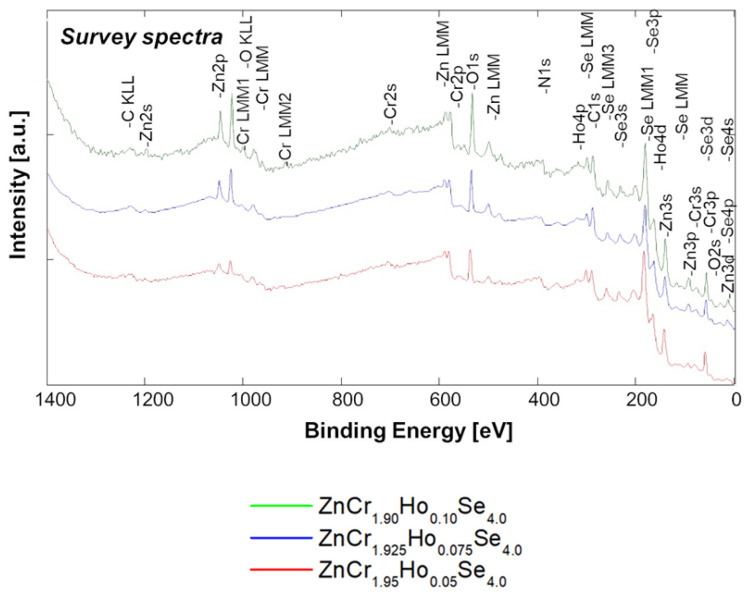
XPS survey spectra of ZnCr_2–x_Ho_x_Se_4_ (x = 0.05, 0.075, and 0.10).

**Figure 16:**
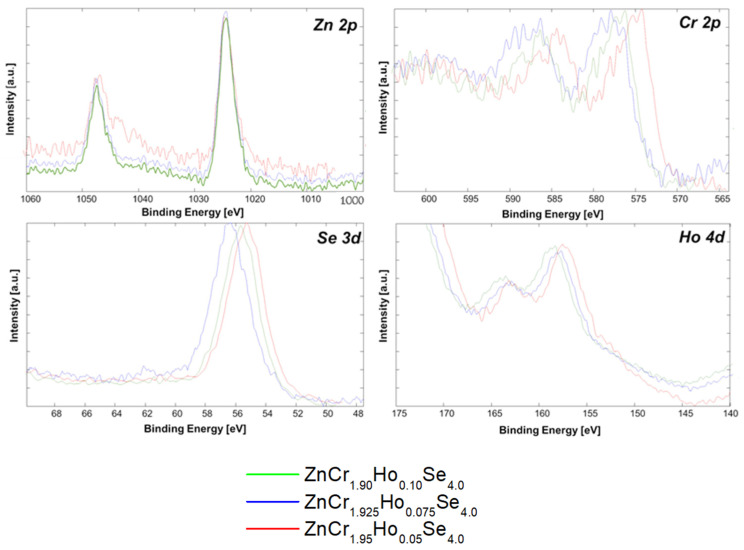
XPS core lines: Zn 2p, Cr 2p, Se 3d, and Ho 4d of ZnCr_1–x_Ho_x_Se_4_ (x = 0.05, 0.075, and 0.10).

The XPS survey spectra were measured from 0 to 1400 eV. They exhibit lines from Zn, Cr, Ho, and Se (Figure 15). Oxygen and carbon ions were observed at the significance level. Still, we eliminated them by sputtering the samples using an Ar+ gun. Apart from the charge states related to the nominal composition of ZnCr_2_HoSe_4_, Ho’s other contributions have been distinguished by broadening the XPS patterns. The *Zn 2p* doublet line shows more than one contribution. The peak position for *Zn 2p_3/2_* is equal to 1022.1 eV for ZnCr_1.9_Ho_0.1_Se_4_, ZnCr_1.925_Ho_0.075_Se_4_, and ZnCr_1.95_Ho_0.05_Se_4_. These small changes in the energy and shape of the Zn 2p_3/2_ and 2p_1/2_ lines correspond with the varying compensation of Zn. The *Cr 2p_3/2_* peak position is equal to approx. 574.7 eV for ZnCr_1.95_Ho_0.05_Se_4_, 576.5 eV for ZnCr_1.9_Ho_0.1_Se_4_, and 577.7 eV for ZnCr_1.925_Ho_0.075_Se_4_. The significant line widening and deformation of *Cr2p_3/2_* and *2p_1/2_* lines can be assigned to the close neighbours of the *Zn LMM* (Auger line) and generally result from significant interactions caused by doping processes. The binding energy of *Ho 4d* is strongly correlated with the locations of substitution of holmium dopants to 157.8 eV for ZnCr_1.95_Ho_0.05_Se_4_, 158.7 eV for ZnCr_1.925_Ho_0.075_Se_4_, or 159.1 eV for ZnCr_1.9_Ho_0.1_Se_4_. The Ho line shows a deformed shape and broadening. High values of the FWHM parameters were connected to the influence of Ho doping and overlapping with *Se LMM* (Auger line) peaks and *Se 3p* in this energy area (Figure 16).

The binding energy of the broad Se 3d line is 54.7 eV for ZnCr_1.95_Ho_0.05_Se_4_, 55.2 eV for ZnCr_1.9_Ho_0.1_Se_4_, and 56.4 eV for ZnCr_1.925_Ho_0.075_Se4, but it is chemically shifted by approximately ~0.9 eV. The binding energy of *O 1s* broad peak is equal to 532.3 eV for ZnCr_1.95_Ho_0.05_Se_4_, 531.8 eV for ZnCr_1.9_Ho_0.1_Se_4_, and 531.4 eV for ZnCr_1.925_Ho_0.075_Se_4_. The binding energy of the *C1s* broad peak is equal to 286.6 eV.

## 3. Materials and Methods

### 3.1. Synthesis of Microcrystalline ZnCr_2−x_Ho_x_Se_4_ Solid Solution

Microcrystalline samples of ZnCr_2−*x*_Ho*_x_*Se_4_ solid solution were obtained by two-step synthesis. In both steps, a high-temperature solid-state reaction between proper reactants was applied. The following high-purity elements: Zn, Ho, Cr, and Se (5N, Sigma Aldrich, St. Louis, MO, USA) were used in synthesis (initial concentration of Ho_2_Se_3_: 2.50, 3.75, and 5.00 mol%). The details of the synthesis are described in Refs. [24,25]. The synthesis of ZnCr_2−*x*_Ho*_x_*Se_4_ (*x* = 0.05, 0.075, and 0.10) materials can be described according to the chemical equation

ZnSe + (1 − *x*/2)Cr_2_Se_3_ + *x*/2 Ho_2_Se_3_ = ZnCr_2−*x*_Ho*_x_*Se_4_. (6)

### 3.2. Experimental Techniques

Characterization of microcrystalline ZnCr_2−*x*_Ho*_x_*Se_4_ samples was performed using the following techniques: X-ray Powder Diffraction (XRPD, PW3050 X’Pert Philips, Malvern Panalytical, Malvern, UK), Scanning Electron Microscopy (Microscope JEM 6480 (JEOL USA, Inc., Peabody, MA, USA) and X-ray Photoelectron Spectroscopy (XPS, Physical Electronics PHI 5700/660 XPS spectrometer, Chanhassen, MN, USA)), simultaneous Differential Scanning Calorimetry and Thermogravimetry (The LabsysEvo, Setaram Inc., Cranbury, NJ, USA), FPLO (Full-Potential Local-Orbital) calculation, and UV–vis–NIR Diffuse Reflectance Spectroscopy (UV–Vis–NIR Spectrophotometer, JASCO-V670, Tokyo, Japan). Magnetic measurements and specific heat capacity, the most essential part of our work, were determined using a QD-PPMS measurement system (Quantum Design Physical Properties Measurement System, Quantum Design, San Diego, CA, USA). We have described these research methods in previous works [24,25,26,28,51,52,55,56]. The effective number of Bohr magneton was calculated from the following formula:

(7) $$p_{eff}=\sqrt{\left(2-x\right)\times p_{Cr}^{2}+x\times p_{Ho}^{2}}$$

where $p=g\sqrt{J(J+1)}$ for the Cr^3+^ ion (*J* = *S* = 3/2, *g* = 2) with 3*d*^3^ and for the Ho^3+^ ion (*J* = 8, *g* = 5/4) with 4*f*^10^ electronic configurations, respectively.

The electronic structure of the ZnCr_1.95_Ho_0.05_Se_4.0_ compound was studied using the FPLO method with the FPLO5-00-20 computer code [58,59,60,61,62]. This relatively old code was used as it allows the study of alloyed systems within the Coherent Potential Approach (CPA) [63], which is crucial in studies where holmium is assumed to substitute the Cr site uniformly. The exchange-correlation potential was used in the form proposed in Refs. [48,49]. Calculations were carried out in a scalar-relativistic, spin-resolved way for 29 *k*-points in a reduced Brillouin Zone (the spinel unit cell is large; therefore, many *k*-points were found to be sufficient). The following approach was chosen: Valence states 4*s*, 4*p*, and 3*d* were considered for Zn, Cr, and Se, and 6*s*, 6*p*, and 5*d* states were considered for Ho. As the semicore states, 3s and 3p were considered for Zn, Cr, and Se, and 5f, 5s, and 5*p* were considered for Ho. Calculations were performed in a spin-resolved, scalar-relativistic way.

## 4. Conclusions

Microcrystalline samples of Ho^3+^-doped spinels with the general formula of ZnCr_2−*x*_Ho*_x_*Se_4_ (where *x* = 0.05, 0.075, and 0.10) were successfully obtained by a high-temperature, two-step reaction in the solid state between adequate reactants. The limited solubility Ho^3+^ in the ZnCr_2_Se_4_ matrix was *x* = 0.10, similar to the microcrystalline ZnCr_2−*x*_Dy*_x_*Se_4_ solid solution.

The significant difference in the ionic radii of Cr^3+^ and Ho^3+^ creates a deformation in the anion octahedron and in the A–X distances, which causes the smaller stabilization of the ionic bond of Cr^3+^ ions and an increase in the ionic bond contribution in the tetrahedral sublattice. In the microcrystalline ZnCr_2−x_Ho_x_Se_4_ spinel, we found a strong influence of holmium ions in octahedral positions on the critical fields connected with increasing the unit cell size and the B-B bonds’ length. For this purpose, X-ray diffraction, Scanning Electron Microscopy, thermal analysis, magnetic and specific heat measurements, and FPLO calculations were performed.

To conclude, the results obtained by the XPS test discerned Ho 4d charge states. XPS experimental results have demonstrated the effects of different concentrations and mixed valence states of Zn and Cr. The deformation and broadening of the core lines correspond to the charge compensation caused by the doping processes. Overlapping lines made it impossible to fit the lines more clearly. Moreover, in magnetic studies, orbital and Landau diamagnetism were estimated, allowing the correct determination of magnetic parameters. The novelty of this work is the elimination of the contribution of magnetic susceptibility, independent of temperature, from the measured total magnetic susceptibility.

## Figures and Tables

**Table 1:** Parameters of the metal ions: electronic configuration, ionic radius (coordination number (CN) in a bracket), electronegativity (Pauling scale), and *p_eff_* (the effective magnetic moment per ion) [35,36,37].

Cation	Electron Configuration	Ionic Radius (Å)	Electronegativity	p_eff_ *	Basic Term
Zn^2+^	[Ar] 3*d*^10^	0.60 ^(CN = 4)^	1.65	0	^1^S
Ho^3+^	[Xe] 4*f*^10^	0.90 ^(CN = 6)^	1.23	10.6	^5^J_8_
Cr^3+^	[Ar] 3*d*^3^	0.62 ^(CN = 6)^	1.66	3.87	^4^F_3/2_

* $p_{eff}=g\sqrt{J\left(J+1\right)}$, where *g* is the Landé factor and *J* is the total angular momentum of the metallic ion.

**Table 2:** Chemical composition of ZnCr_2−*x*_Ho*_x_*Se_4_ materials obtained from SEM studies.

*x*	% Mass				Chemical Composition
	Zn	Cr	Ho	Se	
0.05	13.82(2)	20.52(3)	1.68(2)	63.98(1)	Zn_0.99_Cr_1.95_Ho_0.05_Se_4.0_
0.075	13.83(1)	20.21(2)	2.49(2)	63.47(3)	Zn_1.00_Cr_1.925_Ho_0.0.075_Se_4.0_
0.10	13.79(2)	19.93(1)	3.15(2)	63.13(1)	Zn_0.99_Cr_1.90_Ho_0.10_Se_4.0_

**Table 3:** Parameters determined from DSC-TG analysis of pure ZnCr_2_Se_4_ and ZnCr_2−*x*_Ho*_x_*Se_4_ samples [24].

Formula	Mass Loss (%)	Onset (^o^)	Offset (^o^)	Peak Minimum (^o^)	Peak Height (mW)	Peak Area (J)	Enthalpy (J/g)
ZnCr_2_Se_4_	5	710	740	725	1.66	0.13	20.2
ZnCr_1.95_Ho_0.05_Se_4.0_	34						
I peak		751	786	764	8.13		29.3
II peak		1155	1189	1172	9.61		39.6
III peak		1344	1359	1355	31.8		60.7
ZnCr_1.925_Ho_0.075_Se_4.0_	35						
I peak		732	779	760	9.62		59.5
II peak		1139	1202	1162	11.34		67.8
III peak		1345	1358	1354	35.0		97.5
ZnCr_1.90_Ho_0.10_Se_4.0_	36						
I peak		735	764	749	2.35		9.17
II peak		1151	1192	1170	9.38		74.5
III peak		1343	1357	1353	32.2		62.2

**Table 4:** Magnetic parameters of microcrystalline ZnCr_2−*x*_Ho*_x_*Se_4_ spinels: *C* is the Curie constant, *T*_N_ is the Néel temperature, *θ* is the Curie–Weiss temperature, *µ_eff_* is the effective magnetic moment, *M* is the magnetization at 5 K and 70 kOe, *p*_eff_ is the effective number of Bohr magnetons, *χ_0_* is the slope, *b* is the intercept, *J*_1_ and *J*_2_ are the superexchange integrals for the first two coordination spheres, and *H*_c1_ and *H*_c2_ are the critical fields. Experimental data for ZnCr_2_Se_4_ were compared from Ref. [24].

x	C (emu·K/mol)	T_N_ (K)	θ (K)	µ_eff_ (µ_B_/f.u.)	M_(5K)_ (µ_B_/f.u.)	p_eff_	χ_0_ (emu/mol)	b (emu·K/mol)	J_1_ (K)	J_2_ (K)	H_c1_ (kOe)	H_c2_ (kOe)
0	3.270	21.9	55	5.114	5.74	5.477	−0.001245	3.66828	−2.37	1.01	10.0	65
0.05	4.537	21.3	66	6.024	4.82	5.906	−0.000809	5.25961	−2.10	1.08	11.6	60.7
0.075	4.727	21.7	51	6.069	5.54	6.108	−0.001071	5.21961	−2.41	0.97	8.0	58.2
0.10	4.848	22.3	34	6.227	5.78	6.305	−0.001121	5.31961	−2.78	0.84	9.4	59.5

## Data Availability

Data is contained within the article or Supplementary Material.

## Supplementary Materials

The following supporting information can be downloaded at: https://www.mdpi.com/article/10.3390/ijms25147918/s1.
