# Supplementary material for: Effect of Ho3+ Substitution on Magnetic Properties of ZnCr2Se4
Source: Int J Mol Sci. 2024 Jul 19;25(14):7918. doi: 10.3390/ijms25147918 (PMC11276618; doi:10.3390/ijms25147918)
Supplement: Supplementary file 1 [file ijms-25-07918-s001.zip › ijms-3099740-supplementary.pdf]

Supplementary Materials

Table S1

Structural parameters obtained from the Rietveld refinement for $ZnCr_{2-x}Ho_xSe_4$ solid solution.

Nominal composition	R_p (%)	R_{wp} (%)	R_{exp} (%)	R_B (%)	χ^2	Lattice parameter a (Å)	Anion parameter u
$ZnCr_{1.95}Ho_{0.05}Se_4$	3.62	5.14	2.53	4.87	4.03	10.4986(7)	0.2598(6)
$ZnCr_{1.925}Ho_{0.075}Se_4$	3.77	5.23	2.58	4.89	4.08	10.5112(2)	0.2615(2)
$ZnCr_{1.90}Ho_{0.10}Se_4$	3.83	5.31	2.62	4.91	4.12	10.5245(2)	0.2622(2)

Table S2

Values of atomic coordinates (x, y, z) and site occupancy (g) of microcrystalline $ZnCr_{2-x}Ho_xSe_4$ solid solution.

Atom	Wycoff notation	x = 0.05		x = 0.075		x = 0.10	
		x = y =z	g	x = y =z	g	x = y =z	g
Se	32e	0.2598	0.1670	0.2615	0.1670	0.2622	0.1670
Zn	8a	0.1250	0.0420	0.1250	0.0420	0.1250	0.0420
Cr	16d	0.5000	0.0813	0.5000	0.0802	0.5000	0.0792
Ho	16d	0.5000	0.0021	0.5000	0.0031	0.5000	0.0042

Table S3

Interatomic distances, angles, structure filling coefficient (α), and the ionic packing coefficients (P_t – tetrahedral position, P_o – octahedral position) for samples under investigation and pure $ZnCr_2Se_4$, $\langle R_A \rangle$ and $\langle R_B \rangle$ are the average cation radii in the tetrahedral and octahedral position, respectively [30,31]. The r_t and r_o are the tetrahedral position radius and octahedral position radius, respectively.

Bond distances and angles and structure coefficients	x parameter 0.00	0.05	0.075	0.10
AX_4 (tetrahedron)				
d(A – X) (Å)	2.439	2.446		2.480
X – A – X (°)	109.47	109.47		109.47
$d(X – X)_1$ (Å)	3.975	3.987		4.043
BX_6 (oktahedron)				
d(B – X) (Å)	2.528	2.527		2.513
$d(X – X)_2$ (Å)	3.419	3.413		3.367
$d(X – X)_3$ (Å)	3.714	3.717		3.724
$X – B – X_1$ (°)	87.89	87.32		87.12
$X – B – X_2$ (°)	92.11	92.68		92.88
XAB_3 (polihedron)				
d(A – X) (Å)	2.439	2.446		2.480
d(B – X) (Å)	2.528	2.527		2.513
d(A – B) (Å)	4.348	4.352		4.357
d(B – B) (Å)	3.697	3.700		3.705
d(A – A) (Å)	4.540	4.540		4.546
A – X – A (°)	120.32	121.06		121.35
B – X – B (°)	96.54	96.83		96.95
r_t (Å)	0.459	0.466		0.500
r_o (Å)	0.544	0.542		0.528
P_t	0.765	0.776		0.833
P_o	0.877	0.854		0.814
α (%)	92.12	92.01		91.76

<R_A>(Å)	0.600	0.600	0.600
<R_B>(Å)	0.620	0.635	0.649

Table S4
Cation distribution and X-ray intensity ratio of $ZnCr_{2-x}Ho_xSe_4$ samples.

x	Site occupation		Intensity ratio							
	A	B	I_{220}/I_{444}		I_{400}/I_{440}		I_{444}/I_{422}		O_{hk0}	O_{220}
			calc.	obs.	calc.	obs.	calc.	obs.		
0.05	(Zn)	[$Cr_{1.95}Ho_{0.05}$]	1.45	1.55	1.03	1.14	1.65	1.67	2.02	0.52
0.075	(Zn)	[$Cr_{1.925}Ho_{0.075}$]	1.42	1.37	0.99	1.04	1.46	1.54	2.08	0.55
0.10	(Zn)	[$Cr_{1.90}Ho_{0.10}$]	1.39	1.24	0.91	0.98	1.32	1.43	2.14	0.57

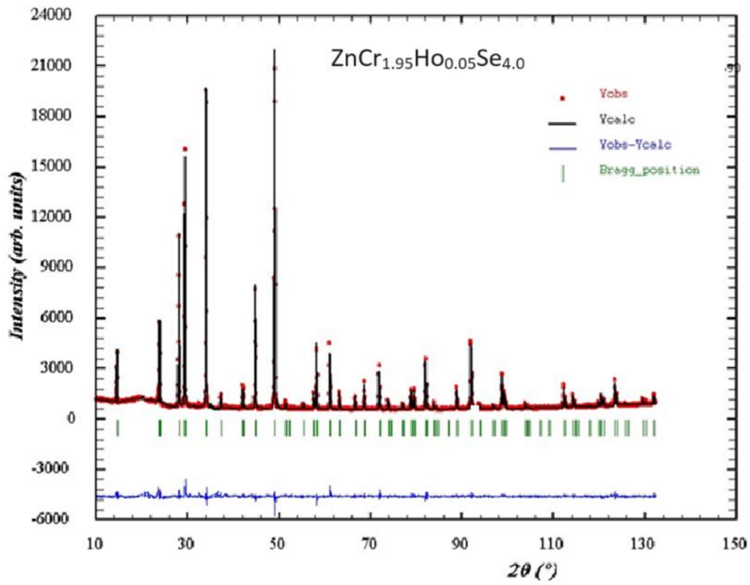

Figure S1. The relation between observed and calculated X-ray diffraction patterns and their difference in the
$ZnCr_{2-x}Ho_xSe_4$ sample when $x = 0.05$.

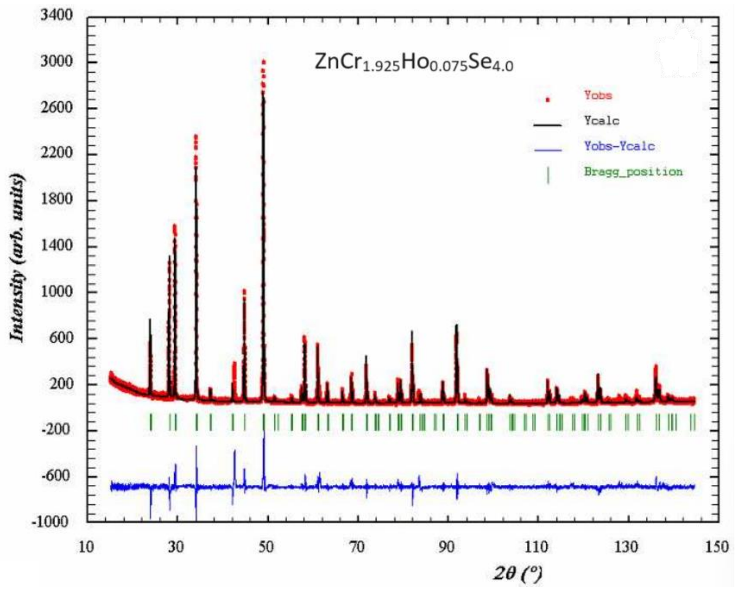

Figure S2. The relation between observed and calculated X-ray diffraction patterns and their difference in the
$ZnCr_{2-x}Ho_xSe_4$ sample when x = 0.075.

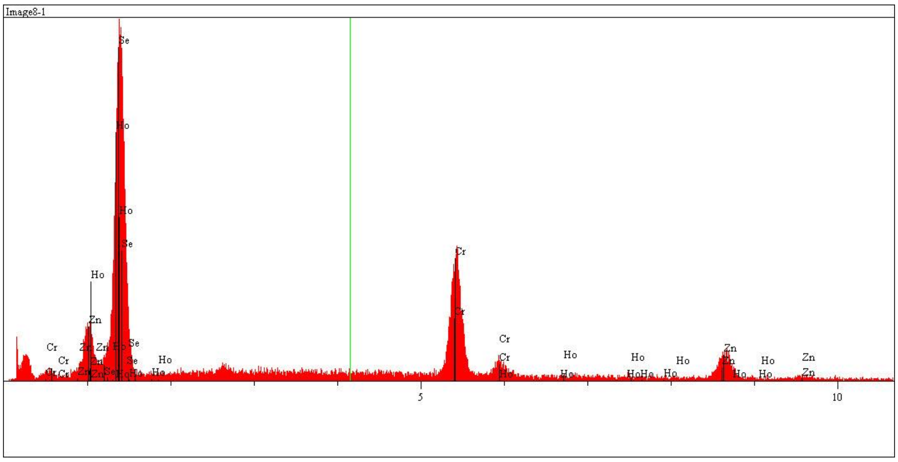

Figure S3. SEM image for $ZnCr_{1.95}Ho_{0.05}Se_4$.

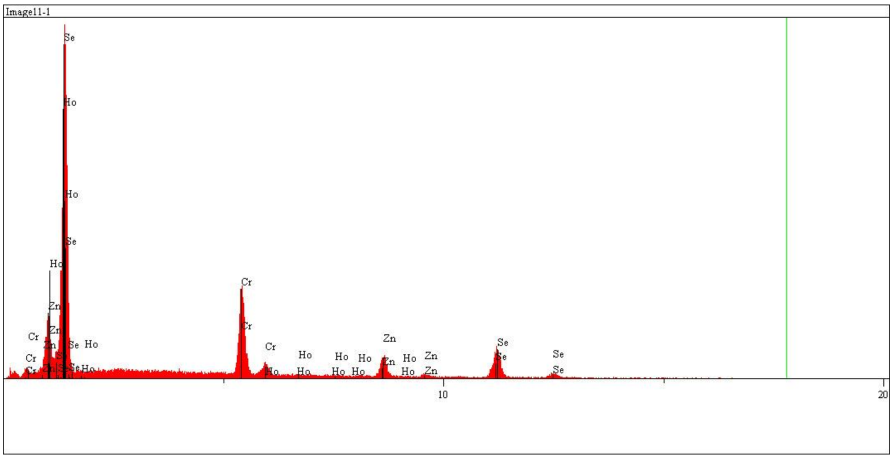

Figure S4. SEM image for $ZnCr_{1.925}Ho_{0.075}Se_4$.

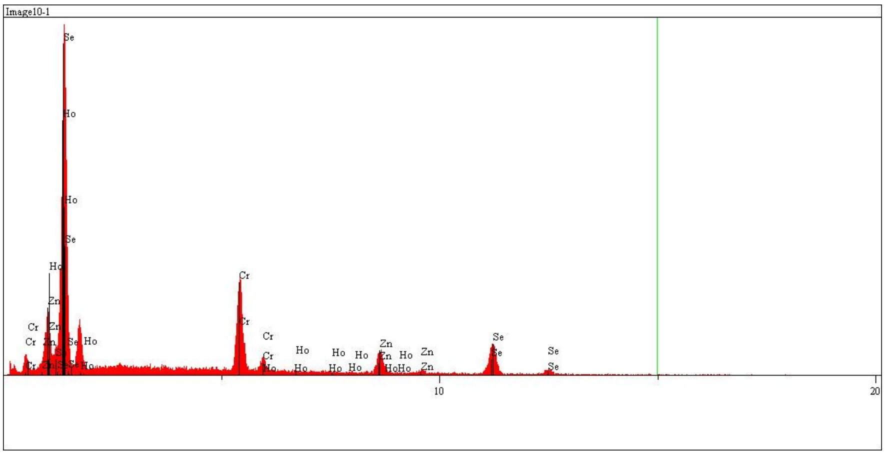

Figure S5. SEM image for $ZnCr_{1.90}Ho_{0.10}Se_4$.
